# A GIS-Aided Assessment of the Health Hazards of Cadmium in Farm Soils in Central Taiwan

**DOI:** 10.3390/ijerph8093759

**Published:** 2011-09-20

**Authors:** Po-Huang Chiang, Ta-Chien Chan, Dennis P. H. Hsieh

**Affiliations:** 1 Division of Health Service Research and Preventive Medicine, Institute of Population Health Sciences, National Health Research Institutes, No. 35, Keyan Road, Zhunan Town, Miaoli County 350, Taiwan; E-Mail: dachianpig@gmail.com; 2 Department of Risk Management, China Medical University, No.91, Hsueh-Shih Road, Taichung 40402, Taiwan; E-Mail: dphsieh@mail.cmu.edu.tw

**Keywords:** kriging, cadmium, soil pollution, health hazard

## Abstract

A geostatistical method was developed to examine the correlation, or lack of it, between the levels of cadmium (Cd) detected in farm soils and those detected in the human specimens collected from residents around the contaminated areas in Changhua County where cadmium contamination of staple rice has been documented. We used the Taiwan EPA environment data in 2002 and human data which were generated by the National Health Research Institutes during 2003–2005. Kriging interpolation methods were used to determine soil Cd concentrations. A Zonal statistical function was performed to assess the individual exposure. Soil Cd levels and tissue Cd levels in residents were analyzed for contamination hotspots and other areas to determine correlation between the two variables. Three Cd contamination hotspots were identified, in which no correlation was found between soil Cd levels and tissue Cd levels in residents. Our results demonstrate how GIS spatial modeling technique can be used to estimate distribution of pollutants in an area using a limited number of data points. Results indicated no association between the soil contamination and the exposure of residents to Cd, suggesting that both the soils and the residents are receptors of Cd as a pollutant from as yet unidentified sources.

## 1. Introduction

Decades of intensive industrial and agricultural practices have left various communities in Taiwan facing potential health threats from pollution. In 2002, the Taiwan Environmental Protection Agency (TEPA) collected 2,251 topsoil samples in four counties in central Taiwan (Changhua, Yunlin, Nantou and Chiayi) and analyzed the levels of Cadmium (Cd) and seven other heavy metals: arsenic (As), copper (Cu), chromium (Cr), mercury (Hg), nickel (Ni), lead (Pb) and zinc (Zn). According to TEPA, 44% of these samples’ concentration exceeded the soil pollution control standard, including 492 farmlands (125.65 ha registered) with a total contaminated farming area of 108.38 ha in Changhua [1]. The results showed that the soil of the investigated area in Changhua County is obviously polluted. Based on a geostatistical study and the distribution of the irrigation channels; the area neighboring the investigated farmland in this project is suspected of being polluted [2].

Among the all heavy metals, cadmium is one of the most reported heavy metals in regards to human health, and has been associated with kidney damage (end stage renal disease), lung cancer, and severe itai-itai–skeletal disease in Japan [3–5]. In order to estimate the health effect from Cd in soil, Geographic Information System (GIS) applications have been shown to be effective in soil prediction [6] and are applied to implement risk assessment between environmental Cd exposure and human health [7]. This study is the first attempt to not only examine the range of possible soil pollution and to identify hazardous areas with more comprehensive modeling techniques, but also to analyze the potential health risk for the inhabitants by spatially matching the residents’ blood, urine and hair Cd levels.

## 2. Methods

The study area is Changhua City and its surrounding towns, which are categorized as a high metal industry area. To address concerns about environmental contamination, topsoil samples (N = 2,743) were collected by TEPA between 2002 and 2004 within Changhua City and its surrounding towns, to provide insights into the potential environmental hazards in the soil. The National Health Research Institute (NHRI) has conducted a large scale investigation since 2002. Residents who lived in this area more than five years were sampled for physical examination. Four hundred and twelve participants were sampled from the high intensity metal industry area in Changhua city and its surrounding area. Blood, urine and hair levels were measured for heavy metal concentration. A spatial merge was performed to merge the interpolated Cd soil surface levels with the participants’ health examination data (N = 412). ArcGIS 9.1 and its extensions were used to perform exploratory spatial data analysis and plot pollution prediction maps. We adopted a standardized geostatistical method [8] by using Ordinary Kriging and Indicator Kriging models (TEPA’s threshold for food crop farmland: 5 mg/kg) to determine the range and distribution of Cd contamination in the study area. A zonal statistic function was performed to calculate possible individual exposures. Pearson’s correlation, one-way analysis of variance (ANOVA) was used to determine the relationship between the human exposure levels and human tissue levels. Buffer analysis was also used to determine the hotspot effect on the human tissue levels. Three distance values (500 m, 1000 m, 1500 m) were set surrounding the hotspots to analyze whether the Cd tissue levels were correlated to the proximity of the hotspots.

## 3. Results and Discussion

**Figure 1:**
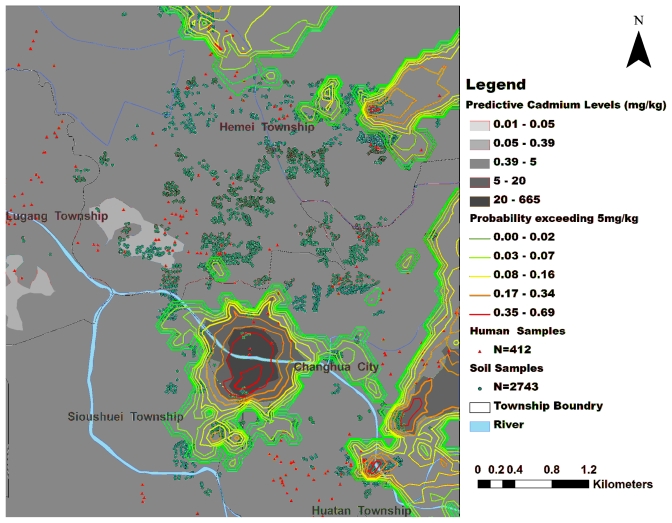
Prediction map of cadmium levels and probability map exceeding the regulatory level.

Maps representing the best fit interpolation methods for Cd are included in Figure 1. It identified hotspots in the prediction map that also had higher probability of exceeding the regulatory soil levels.

There was no significant correlation between soil Cd levels and human tissue levels. Hotspot analysis was used to determine if the distance away from the hotspot would have different impact on the tissue levels, but no significant difference among three distance groups was found (Table 1). Nevertheless, the people who had higher Cd levels in their tissues were located outside the soil hotspots. The Cd levels of blood was borderline significant (p = 0.05) higher in the buffer of 1500 m.

This study is the attempt try to correlate the Cd exposure levels from topsoil with the blood and urine levels from the local residents. Ordinary Kriging resulted in three Cd hotspots (clustering of high level of contaminations) in the southwestern region of the study area (See Figure 1) with a range of 273.05 to 665.00 mg/kg – substantially above every class set by TEPA. Blood and urine Cd levels indicated that the high concentration in tissue levels that was not located in the hotspot area. Although this project showed no evidence of health hazard by Cd due to topsoil exposure, the interpolation methods and buffer analysis were demonstrated to be feasible risk assessment tools.

The residents in this high industrial area did show some degree of higher levels of Cd exposure than low industrial area ones. High levels of Cd was found in their hair, an indicator of long-term exposure; while above threshold tissue levels, which may be due to factory exposure or food consumption, were also found. There are three exposure pathways of Cd including inhalation, oral, and dermal. This study was tried to link between the soil exposure and possible orally intake in one time point. The cumulative health effect could not be reflected by our cross-sectional study design. All levels exceed the OSHA action level and needs some attention. Even though we didn’t find any significant direct correlation between soil levels and tissue levels, there is a need to pay attention on the health of residents living in such highly heavy metal polluted areas.

## 4. Limitations

As with all the models, assumptions due to the limited data available create several limitations in this analysis. There were not enough human samples in the hotspot area to provide enough evidence for exposure assessment. Lack of a control group to compare with the exposed human sample is also a limitation for this study. Different exposure media and pathways, like irrigation channels, rivers or occupational exposures, also need to be examined to determine the health risk in metal industry areas. There were still many confounders in evaluating health hazard such as age, sex, smoking and occupation, etc. In future work, a geographically weighted regression could be applied to both individual confounders and spatial factors at the same time.

## Figures and Tables

**Table 1:** Tissues levels in different buffer distances.

	Cases in different buffer distances			
	500 m	1000 m	1500 m	

Cd in different tissues	N = 51	N = 34	N = 85	p-value
Urine (μg/L)	1.21 (SD = 0.94)	1.14 (SD = 0.89)	1.24 (SD = 1.01)	0.88
Blood (μg/L)	1.50 (SD = 0.69)	1.43 (SD = 0.81)	1.86 (SD = 1.29)	0.05 *
Hair (μg/kg)	0.11 (SD = 0.20)	0.11 (SD = 0.25)	0.12 (SD = 0.24)	0.94

* p < 0.05
